# Prophylactic regimens for the prevention of pseudophakic cystoid macular edema: systematic review and meta-analysis

**DOI:** 10.1186/s40942-024-00588-8

**Published:** 2024-10-10

**Authors:** Abdullah S. Alqahtani, Reem M. Hersi, Jumana J. Homsi, Loujen O. Alamoudi, Sara Alghamdi, Rawan K. Alrajhi, Reham A. AlJehani

**Affiliations:** 1 Department of Surgery, Division of Ophthalmology, King Abdulaziz Medical City, Ministry of National Guard Health Affairs, Jeddah, Saudi Arabia; 2 King Saud bin Abdulaziz University for Health Sciences, Jeddah, Saudi Arabia; 3 King Abdullah International Medical Research Center, Jeddah, Saudi Arabia; 4 Department of Ophthalmology, King Khaled Eye Specialist Hospital, Riyadh, Saudi Arabia; 5 Ophthalmology Residency Training Program in Western Region, King Abdulaziz University Hospital, Jeddah, Saudi Arabia; 6 Ophthalmology Department, King Faisal Specialist Hospital and Research Center, Riyadh, Saudi Arabia; 7 Ophthalmology Residency Training Program in Western Region, Jeddah, Saudi Arabia; 8 Department of Ophthalmology, Makkah Health Cluster, Makkah, Saudi Arabia; 9 Department of Ophthalmology, King Abdulaziz University, Jeddah, Saudi Arabia; 10 Ophthalmology Residency Training Program in Western Region, Jeddah, Saudi Arabia

**Keywords:** NSAID, Steroid, Pseudophakic cystoid macular edema

## Abstract

**Background:**

Pseudophakic cystoid macular edema (PCME) is a known complication of cataract surgery that contributes to decreased visual acuity. Mechanical manipulation associated with the release of inflammatory mediators is the leading hypothesis for PCME. To date, no standardized prophylactic protocol has been established to effectively reduce the incidence of PCME. This study assessed the efficacy and safety of nonsteroidal anti-inflammatory drops (NSAIDs) and corticosteroids for the prevention of PCME.

**Method:**

We searched the following databases MEDLINE, EMBASE, and Cochrane Central. Register of Controlled Trials and included randomized controlled trials (RCTs) that studied the efficacy of NSAID vs. placebo, NSAID vs. steroid, or NSAID + steroid vs. placebo, reporting the incidence of PCME, macular thickness, and best-corrected visual acuity. The risk ratio (RR) with a 95% confidence interval (CI) and a random-effects model was used. The risk of bias was assessed using the revised Cochrane risk-of-bias tool.

**Results:**

A total of 18 RCTs were included in this study (*n* = 2959). Nine RCT showed low risk of bias, 7 RCT showed unclear risk of bias, and 2 RCT had high risk of bias. The incidence of cystoid macular edema among patients treated with NSAIDs was significantly lower (RR = 0.33, *P* < 0.001). Subgroup analysis revealed a statistically significant low risk of edema among patients treated with NSAIDs alone (*P* < 0.001) compared to others. NSAIDs were associated with significantly low mean corrected visual acuity values using LogMar (*P* < 0.001).

**Conclusion:**

NSAID alone or in combination with steroids showed its efficacy in reducing the incidence of PCME post-operatively. Future double-blind randomized controlled trials are required to standardize the protocol for different patient population.

**Supplementary Information:**

The online version contains supplementary material available at 10.1186/s40942-024-00588-8.

## Background

Cystoid macular edema (CME) is a well-known postoperative complication characterized by central subfield macular thickening, cystic hyporeflective lesions, and subfoveal fluid when analyzed with optical coherence tomography (OCT) [1]. Pseudophakic CME (PCME, also termed “Irvine-Gass syndrome”), refers to a CME that occurs after cataract surgery. It is considered the most common cause of postoperative visual deterioration [2, 3]. The incidence of PCME varies from 1 to 30%, owing to different definitions and diagnostic criteria. The incidence of clinical PCME in low-risk patients varies from 0.1 to 2.35% [2].

However, its pathophysiology remains unclear. The surgical manipulation within the anterior chamber may lead to the release of arachidonic acid from the uveal tissue, with the production of leukotrienes and prostaglandins [4]. Subsequently, inflammatory mediators diffuse into the vitreous humor and disrupt the blood-retinal barrier, resulting in enhanced vascular permeability and the development of macular edema [4]. Factors associated with an increased risk of PCME are systemic conditions such as age and arteriosclerotic vascular disease, and ocular conditions such as uveitis, diabetic retinopathy (DR), previous diagnosis of epiretinal membrane, retinal vein occlusion, and retinal detachment repair. Surgery-associated factors include trauma during surgery, posterior capsule rupture, vitreous loss, vitreous traction, phacoenergy, and a long duration of surgery [1]. 

The initial treatment includes the use of topical nonsteroidal anti-inflammatory drugs (NSAIDs), either as monotherapy or in combination with topical corticosteroids [5]. Alternative treatments for refractory cases include sub-Tenon’s or intravitreal corticosteroid injections to inhibit arachidonic acid release [4]. Previous studies have extensively reviewed prophylactic regimens to prevent PCME. One of which is PREvention of Macular Edema after cataract surgery (PREMED) study that demonstrated the superiority of combination therapy involving NSAIDs and steroids in preventing PCME [1]. Presently, there is no standardized treatment or prophylactic protocol for PCME prevention and treatment, owing to the lack of strong randomized double-blind placebo trials and comparative studies [2]. This systematic review compared the efficacy of NSAIDs and corticosteroids in reducing postoperative inflammation and preventing PCME.

## Methodology

We completed our systematic review in accordance with the Preferred Reporting Items for.

Systematic Reviews and Meta-Analyses guidelines [6] and a pre-specified protocol registered in PROSPERO (CRD42023414465).

### Eligibility criteria

This systematic review and meta-analysis included all randomized clinical trials (RCTs) that assessed the efficacy of topical NSAID or NSAID + steroid in comparison to steroid alone or placebo in preventing CME after phacoemulsification and intraocular lens insertion. Patients who had undergone extracapsular cataract surgery were excluded. Trials in which the patients had previous maculopathies, Diabetic Retinopathy (DR), or any ocular disease were excluded from the systematic review and meta-analysis. All the editorials, conferences, commentaries, letters to editors, and reviews were excluded from the study. Additionally, non-English studies, non-RCTs, and single-arm studies were excluded.

### Search strategy

The meta-analysis was conducted by searching MEDLINE, EMBASE, and Cochrane Central. Register of Controlled Trials databases for relevant articles published from the date of database establishment to April 18, 2023, using Medical Subject Headings keywords, as outlined in Supplementary Materials. This study had limitations in terms of language but no limitation in regards to date. Duplicate findings were excluded after the search was completed. The references of related articles were retrieved for additional publications that were not found during the systematic search.

### Data extraction

Both the reviewers independently assessed the studies identified in the database search for relevance from the titles and abstract. Articles that potentially met the eligibility criteria were. retrieved. Then the reviewers assessed retrieved studies for inclusion and extracted data including study characteristics and outcome data. Subsequently, the same studies were compared and revised by the two authors. Discrepancies were resolved by discussion with a third reviewer. A customized form, including the following items was used for data extraction: [1] study characteristics, including the first author, year of publication, and sample size; [2] patient characteristics, including mean age, sex, ethnicity, systemic risk factors; [3] intervention characteristics, including the type of intervention, dose, route, and duration; and [4] main outcome measures, including the incidence of CME and secondary outcome measures including best corrected visual acuity, intraocular pressure, anterior chamber cell count, central macular thickness, macular volume, and postoperative complications. Our study aimed to assess the outcome of central retinal thickness; however, relevant literature reviews did not yield sufficient data on this aspect.

### Risk of bias assessment

The quality of the included studies was evaluated independently by the two authors using the revised Cochrane risk-of-bias tool [7]. The overall risk of bias was categorized as “low risk of bias,” “some concerns,” or “high risk of bias,” based on the following five domains: [1] the randomization process [2], deviations from the intended intervention [3], missing outcome data [4], measurement of the outcome, and [5] selection of reported results. Disagreements were resolved through discussions.

### Meta-analysis

Review Manager version 5.4 (Cochrane Collaboration) and Comprehensive Meta-Analysis v3 software were used to analyze the data. The weighted mean difference or standardized mean difference (SMD) was used for analyzing the continuous variables. Data are reported as medians and the range, mean, and range were converted to mean and standard deviation. The risk ratio (RR) with a 95% confidence interval (CI) was used to analyze the binary variables. The fixed-effects model was used when homogeneity between the effect sizes was revealed. Paradoxically, a random-effects model was used once statistical heterogeneity was established. Statistical heterogeneity was determined using the Higgins I^2^ statistic > 50% and Cochrane Q (Chi-square test) at a value of *P* < 0.10 [8]. The statistical significance was set at *P* < 0.05.

## Results

**Fig. 1:**
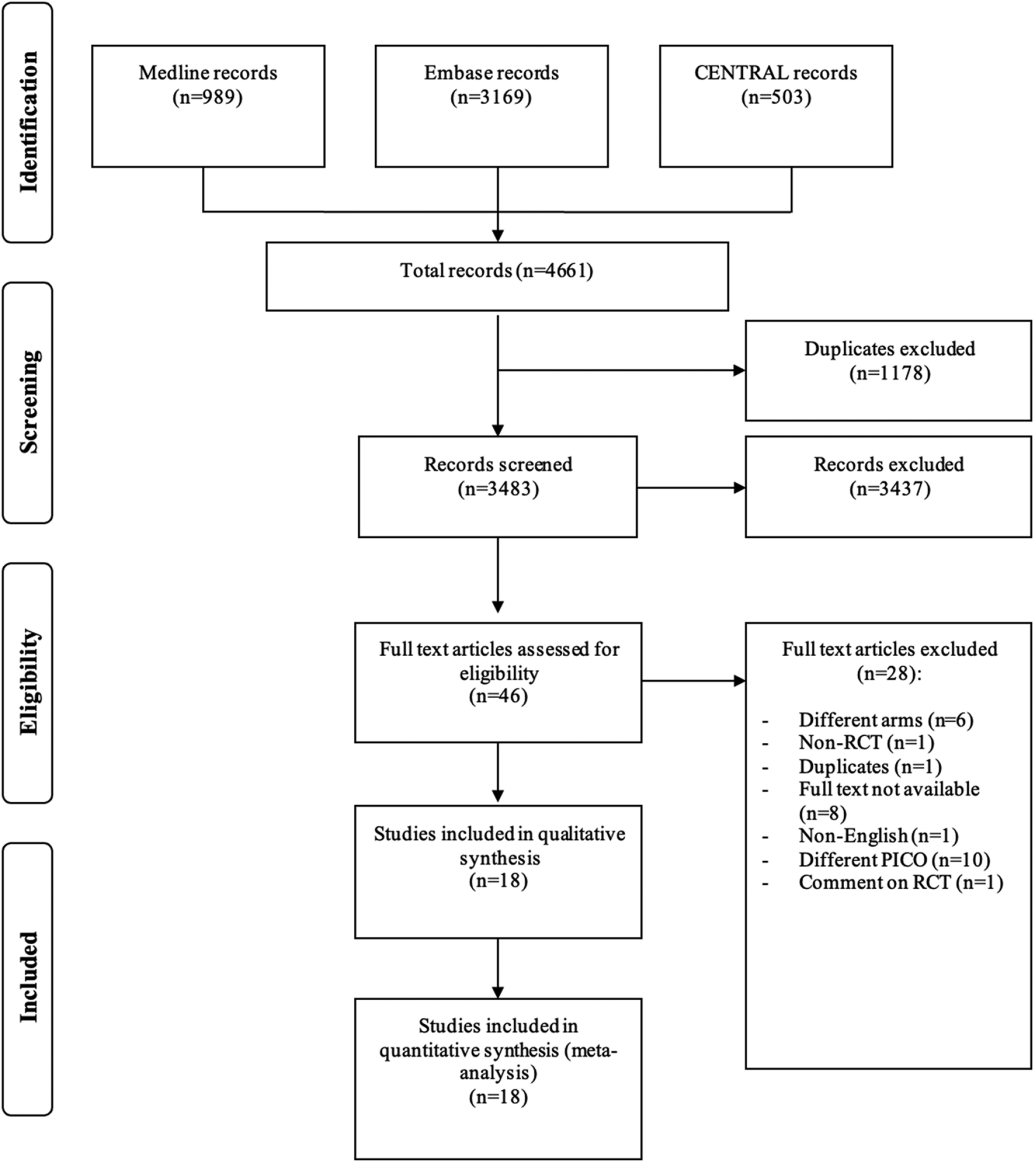
Flowchart of the inclusion and exclusion process

Figure 1 illustrates the flowchart of the study’s inclusion and exclusion processes. A total of 4,661 studies were retrieved from these databases. A total of 1,178 records were duplicates and were initially excluded. After title and abstract screening, 3,437 studies were identified and excluded due to different study designs or different topic, and the remaining 46 underwent full-text screening. Ultimately, 18 studies were included in the meta-analysis.

### Demographic characteristics

This study included 18 articles, encompassing 2,959 patients with cataract. Of these, 1,422 patients received NSAIDs alone and 378 patients received NSAID + steroid (intervention groups), and 1,159 patients received either steroid alone or placebo (control groups). The most administered NSAIDs were nepafenac, followed by ketorolac and diclofenac. Steroids alone were administered to the majority of the control arms, with only five studies administering a placebo. The route of drug administration was topical. The average age of the patients ranged from 60.83 to 76.71 years. The study included 1,091 men and 1,249 women. The average follow-up period ranged from 1 to 3 months (Table 1).

**Table 1:** Demographic characteristics of the included studies

Study ID		Intervention	Control	Intervention-related data		Sample Size		Type of cataract	Age (Years)		Gender		Follow-up periods		Definition of CME
						Intervention	Control		Intervention	Control	Males	Females	Intervention	Control	
				Dose of intervention	Route	Number	Number		Mean ± SD	Number	Number	Number			
1	Singhal et al., [22]	NSAID	Prednisolone 1%	preservative-free ketorolac (0.4%), bromfenac (0.07%), nepafenac (0.3%), topical nepafenac (0.1%)	Topical drops	379	91	All grades of cataract	NR	65.4	271	199	1 and 6 weeks after surgery.		NR
2	Wang et al., [19]	NSAID	Dexamethasone 0.1%	bromfenac sodium 0.1%	Topical drops	116	41	NR	46–92 years, Nebafenac 73.37 ± 9.17)		111	129	Baseline, day 1, week 1, 1 month, 2 months		
3	Stock et al., [9]	NSAID	Propylene glycol	0.3% nepafenac, 0.5% ketorolac tromethamine	Topical drops	53	24	NR			NR	NR	1, 7, and 45 days postoperatively		NR
4	Campa et al., [12]	NSAID + dexamethasone	Dexamethasone	Nepafenac + Dexa, Bromfenac + Dexa	Topical drops	96	48	senile cataract except extremely dense cataracts	nepafenac: 77 ± 5.93, bromfenac: 78.21 ± 7.87		43	53	Baseline, week 1, week 5	Baseline, week 1, week 5	NR
5	Wielders et al., [13]	NSAID	Dexamethasone 0.1%	bromfenac 0.09%, Brom + DEXA	Topical drops	307	Control group 1: 304 Control group 2: 303	Nuclear, Cortical, Subcapsular	bromfenac: 69.70 ± 8.94, brom + dexa: 70.41 ± 8.91	71.23 ± 8.73	NR	NR	Baseline, week 6, week 12	Baseline, week 6, week 12	Cystoid macular edema was defined as an increase in central subfield mean macular thickness of 10% or more over baseline, CSME was defined as CME with less than a 0.2 logMAR CDVA improvement compared with the preoperative baseline. withcysticchangesonSD-OCT.
6	Mathys et al., [15]	NSAID + dexamethasone	Dexamethasone	nepafenac 0.1%	Topical drops	39	40	NS, PSCC, CC	73.95	70.33	37	42	1 day 1 week 1 month, 2 months	1 day 1 week month, 2 months	an average increase in foveal thickness of 10–22 (+/- 24) microns occurs after an uncomplicated phacoemulsification, an increase in CMT by 40 microns or more on OCT was considered to be significant and taken as a criterion for analysis.[21] Clinical CME was defined as a significant increase in CMT along with visible cystic changes and final BCVA less than 6/9.
7	Tzelikis et al., [18]	NSAID	Placebo	nepafenac 0.3%	Topical drops	103	103	Nuclear opalescence	68.32 + 9.08	68.32 + 9.08	39	73	1 week, 5 weeks, 12 weeks.	1 week, 5 weeks, 12 weeks.	Clinically significant macular edema, which consists of the presence of macular edema associated with reduced visual acuity, It is thought to be caused by the release of inflammatory mediators such as prostaglandins and leukotrienes during cataract sur- gery, which can lead to an increase in vascular permeability in the blood–retinal barrier with subsequent accumulation of fluid in the macula.
8	Zaczek et al., [27]	NSAID + dexamethasone	Dexamethasone 0.1%	nepafenac 0.1% and dexamethasone 0.1%.	Topical drops	75	77	NR	70.4 ± 7.4	68.3 ± 7.5	54	98	1 day, 3 weeks, and 6 weeks	1 day, 3 weeks, and 6 weeks	an accumulation of extracellular intraretinal fluid in the outer plex- iform and in the inner nuclear layers of the retina resulting from a breakdown of the blood–retinal barrier in response to a postoperative inflammation in the anterior chamber.
9	Erichsen et al., [14]	NSAID + prednisolone	Pre-treatment	ketorolac tromethamine, 0.5%	Topical drops	94	94	NR	G1: 72.3. G2: 71.8. G3: 72.2. G4: 71.8	72.6	180	290	at the preoperative visit (baseline) and 3 days, 3 weeks, and 3 months postoperatively	at the preoperative visit (baseline) and 3 days, 3 weeks, and 3 months postoperatively	PCME is caused by the in- flammatory response after cataract surgery, which disrupts the blood-ocular barrier leading to leakage of fluid into the retina.4
10	Donnenfeld et al., [13]	NSAID	Placebo	ketorolac tromethamine 0.4%	Topical drops	75	25	NR	72.8 ± 8.5	72.8 ± 8.5	NR	NR	1 day, 2 weeks, and 3 months	1 day, 2 weeks, and 3 months	Cystoid macular edema, a cystic accumulation of extracellular intraretinal fluid in the outer plexiform and inner nuclear layers of the retina, is a result of breakdown of the blood–retinal barrier.
11	Yavas et al., [21]	NSAID	topical steroids and antibiotics	indomethacin 0.1%.	Topical drops	121	58	NR	Group1: 65.28 ± 9.90. Group2: 62.25 ± 11.57	64.78 ± 9.18	106	73	3 moths post-op	3 months post-op	Cystoid macular edema is related to the disruption of the blood–retinal barrier and blood–aqueous barrier (BAB) and the inflammation induced by prostaglandins or other inflammatory mediators.
12	Almeida et al., [11]	NSAID	Placebo	Ketorolac 5%, Nepafenac 5%	eye drops	108	54	NR	2.4 ± 8.2		74	88	At day 0 or 1, at 1 month	At day 0 or 1, at 1 month	NR
13	Ticly et al., [17]	NSAID	Placebo	NR	eye drops	37	44	Nuclear cataract density of 2&3	67.1+/-10.8	66.1 ± 8.7	43	38	Patients were evaluated at 1 day and 5 weeks postoperatively.	Patients were evaluated at 1 day and 5 weeks postoperatively.	Was defined as the presence of well-defined cystic fluid pockets, which presented as hyporeflective lacunae with well-defined boundaries observed in the retina layers17 or a CSF thickness above 315 mm
14	Miyake et al., [16]	NSAID	Dexamethasone 0.1%	Diclofenac 0.1%, fluorometholone 0.1%	eye drops	25	25	Senile	65.4+/-7.0	65.8 ± 7.1	23	27	At2 days, 1 week, 2 weeks,5 weeks	At2 days, 1 week, 2 weeks,5 weeks	captured by fluorescein angiography at 2 and 5 weeks after surgery. It was garded as followes: 0 = no dye accumulation or leakage. 1 = slight dye accumulation at the cystic space & incompletely surrounding the fovea. 2 = dye accumulation surrounding the fovea & a diameter of less than 2 mm. 3 = dye accumulation surrounding the fovea & a diameter greater than 2 mm.
15	Jung et al., [26]	NSAID	Steroid	Bromofenac 0.1%, Ketorolac 0.45%	eye drops	60	31	NR	(G1: 66.9+/-11.1. G2: 67.5+/-7.0. age (range)(year)	66.8 ± 8.1	41	50	Post-op day 1, 7, 28.	Post-op day 1, 7, 28.	CME was defines as prescenec of cystoid changes associated with substantial (> 40 μm) retinal thickness.
16	Howaidy et al., [24]	NSAID	Placebo	Nepafenac: 0.1%	Ophthalmic solution	38	41	cataract density G II or III determined by LOCS III classification	63.1 ± 3.9 (57–69)	62.5 ± 5.3 (51–72)	42	37	1 week, 1 month, and 3 months after surgery	1 week, 1 month, and 3 months after surgery	NR
17	Ibrahim et al., [25]	NSAID + dexamethasone	Dexamethasone 0.1%	Nepafenac: 0.1%	eye drops	36	18	Senile	60.83 ± 4.06 years	60.83 ± 4.06 years	NR	NR	day 1, 1 week and 1 month	day 1, 1 week and 1 month	NR
18	Moschos et al., [23]	NSAID + dexamethasone	Dexamethasone	chloramphenicol 0.5%/dexamethasone sodium phosphate 0.1%/ diclofenac sodium 0.1%	Topical	38	41	NR	76.68 ± 10.72	76.71 ± 8.82	27	52	postoperative days 1, 14, and 28		NR

Abbreviations; NSAIDs = Non-steroidal Anti-inflammatory drugs, SD = standard Deviation, NR = Non-reported, CME = Cystoid Macular edema

### Risk of bias assessment

**Fig. 2:**
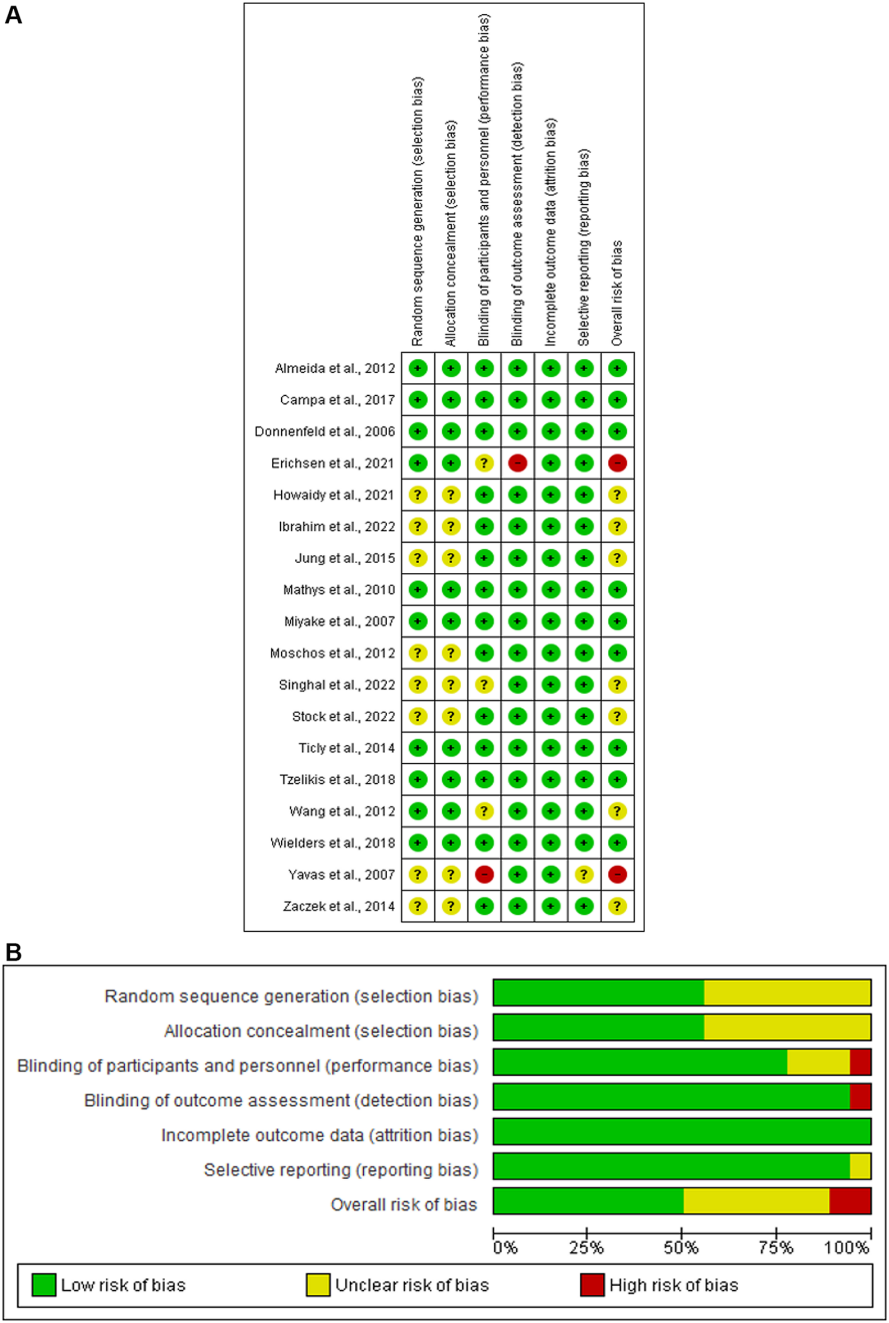
(**A**) Risk of bias graph (**B**) Risk of bias summary: review authors’ judgements about each risk of bias item presented as percentages across all included studies

The risk of bias in the included RCTs was evaluated using the Cochrane Collaboration tool. This tool comprises the following seven items: random sequence generation, allocation concealment (selection bias), participant blinding and personnel performance bias, blinding of the outcome assessment (detection bias), incomplete outcome data (attrition bias), selective reporting (reporting bias), and other possible causes of bias [10]. Ten articles had a low risk of random sequence generation and allocation concealment bias [11–20]. Yavas et al., 2007 showed a high risk of performance bias [21], whereas Erichsen et al., 2021; Wang et al., 2012; and Singhal et al., 2022 revealed an unclear risk [14, 19, 22]. All included studies showed a low risk of detection bias, in addition to Erichsen et al. study (2021) [14]. All included studies showed a low risk of attrition bias, whereas the study by Yavas et al., 2007 showed an unclear risk of reporting bias [21]. Nine articles showed a low overall risk of bias [11–13, 15–18, 20, 23], while two studies showed a high risk of bias [14, 21] (Fig. 2a and b).

### Cystoid macular edema

**Fig. 3:**
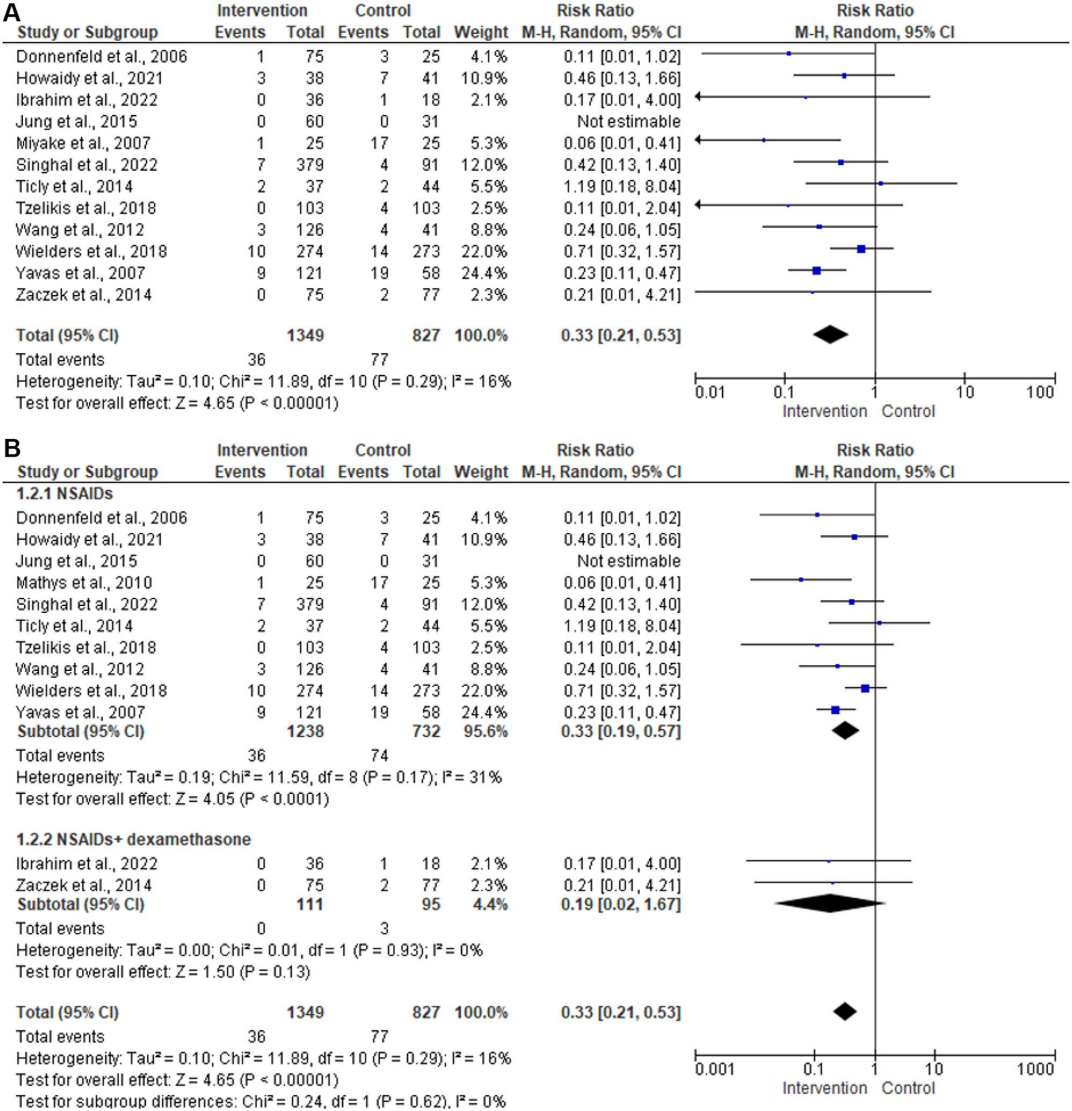
(**A**) Forest plot of summary analysis of the risk ratio (RR) and 95% CI of the risk of macular edema between the NSAIDs group and control group (**B**) Forest plot of the subgroup analysis of the risk ratio (RR) and 95% CI of the risk of macular edema between the NSAIDs group and control group based on the type of the intervention. The size of the blue squares is proportional to the statistical weight of each trial. The black diamond represents the pooled point estimate. The positioning of both diamonds and squares (along with 95% CIs) beyond the vertical line (unit value) suggests a significant outcome (IV = inverse variance)

**Fig. 4:**
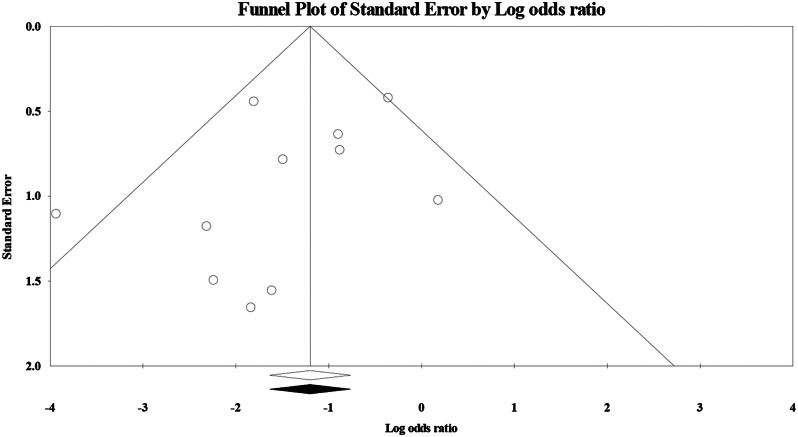
Funnel plot of the publication bias showed symmetrical distribution of studies along the middle line

Twelve studies including 2,179 patients evaluated the risk of CME among those treated with NSAIDs [13, 16–22, 24–27]. In the random-effects model (I^2^ = 16%, *P* = 0.29), the risk of clinical macular edema among patients treated with NSAIDs was significantly low (RR 0.33; 95%CI 0.21–0.53; *P* < 0.001). Subgroup analysis based on the intervention revealed a statistically significant low risk of edema among patients treated with NSAIDs alone (RR 0.33; 95%CI 0.19–0.57; *P* < 0.001). No evidence of publication bias was detected by the symmetrical distribution of studies along the middle line of the funnel plot and based on Egger’s regression test (Intercept = -1.05, *P* = 0.24) (Figs. 3 and 4).

### Central macular thickness

**Fig. 5:**
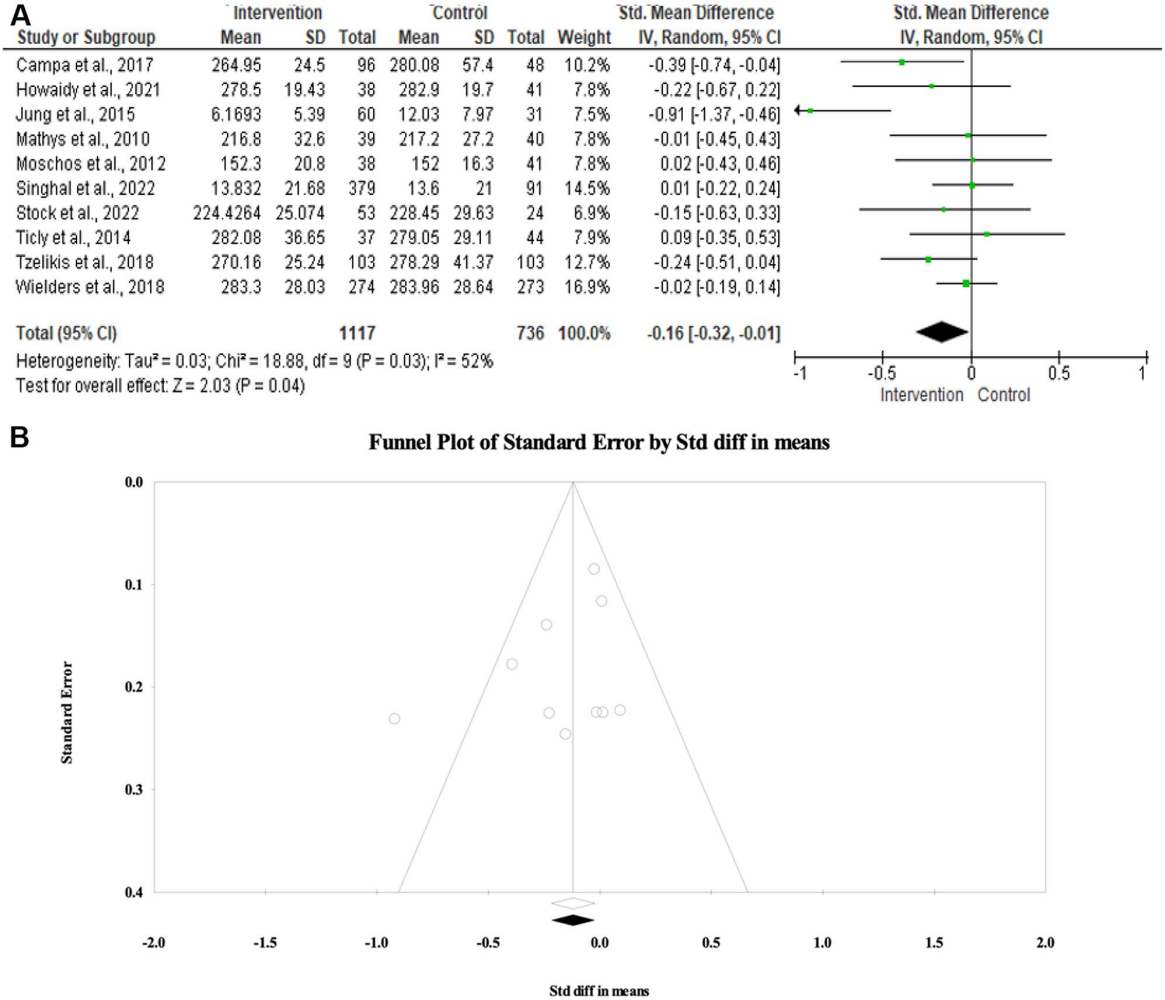
(**A**) Forest plot of summary analysis of the standardized mean difference (SMD) and 95% CI of the central macular thickness between the NSAIDs group and control group. The size of the green squares is proportional to the statistical weight of each trial. The black diamond represents the pooled point estimate. The positioning of both diamonds and squares (along with 95% CIs) beyond the vertical line (unit value) suggests a significant outcome (IV = inverse variance) (**B**) Funnel plot of the publication bias showed symmetrical distribution of studies along the middle line

The mean difference in central macular thickness between the NSAID and control groups was evaluated in 1853 patients. Pooling of data in the random-effects model (I^2^ = 52%, *p* = 0.03) revealed a statistically significant low mean central macular thickness among patients treated with NSAIDs compared to steroid alone or placebo (SMD − 0.16; 95%CI -0.32 to -0.01; *p* = 0.04). No evidence of publication bias was detected by the symmetrical distribution of studies along the middle line of the funnel plot and based on Egger’s regression test (Intercept= -1.5, *p* = 0.24) (Fig. 5).

### Corrected visual acuity

**Fig. 6:**
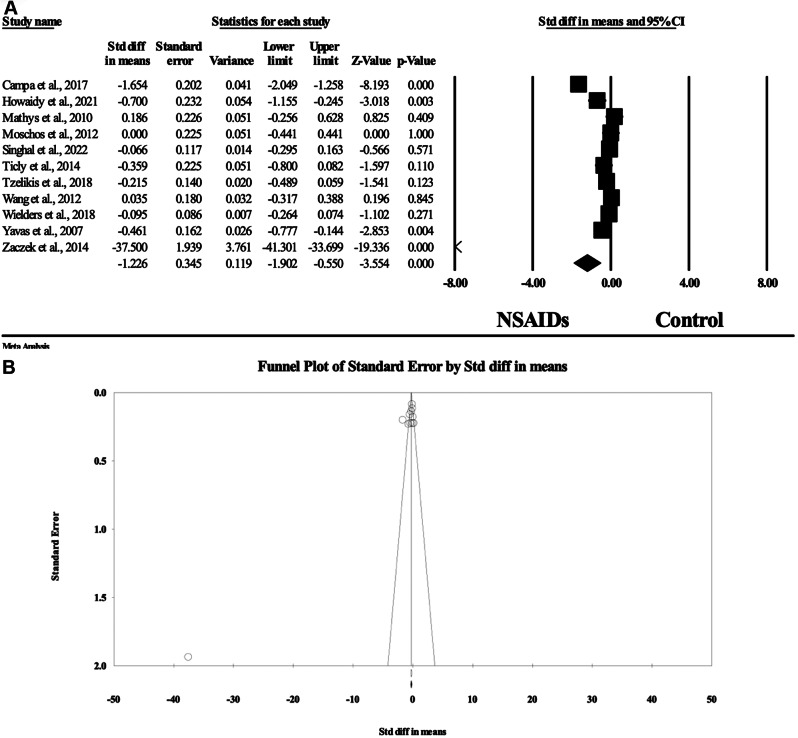
(**A**) Forest plot of summary analysis of the Standardized Mean Difference (SMD) and 95% CI of the mean corrected visual acuity values between the NSAIDs group and control group. The size of the black squares is proportional to the statistical weight of each trial. The black diamond represents the pooled point estimate. The positioning of both diamonds and squares (along with 95% CIs) beyond the vertical line (unit value) suggests a significant outcome (IV = inverse variance) (**B**) Funnel plot of the publication bias showed symmetrical distribution of studies along the middle line

Eleven articles including 2209 patients assessed the difference in the mean corrected visual acuity values between the NSAID and control groups [12, 15, 17–24, 27]. There was a statistically significant lower mean corrected visual acuity values using logMAR among patients treated with NSAIDs with an SMD of -1.226 and 95%CI ranging from − 1.902 to -0.55 in the random-effects model (I^2^ = 97.7%, *p* < 0.001) compared to steroid alone or placebo. No evidence of publication bias was detected by the symmetrical distribution of studies along the middle line of the funnel plot and based on Egger’s regression test (Intercept = -10.35, *p* = 0.015) (Fig. 6).

### Foveal thickness

**Fig. 7:**
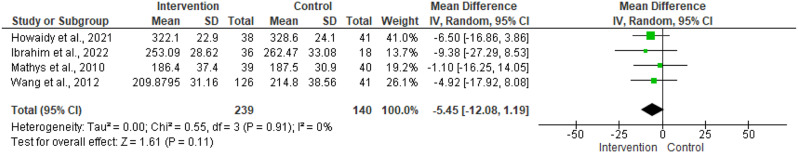
Forest plot of summary analysis of the mean difference (MD) and 95% CI of the mean foveal thickness between the NSAIDs group and control group. The size of the green squares is proportional to the statistical weight of each trial. The black diamond represents the pooled point estimate. The positioning of both diamonds and squares (along with 95% CIs) beyond the vertical line (unit value) suggests a significant outcome (IV = inverse variance)

The difference between the NSAID and intervention groups regarding the mean foveal thickness was evaluated in 379 patients in four articles [15, 19, 24, 25]. There was no statistically significant difference between the groups (MD -5.45; 95%CI -12.08 to 1.19; *p* = 0.11) in the random effects model (I^2^ = 0%, *p* = 0.91) (Fig. 7).

### Intraocular pressure

**Fig. 8:**
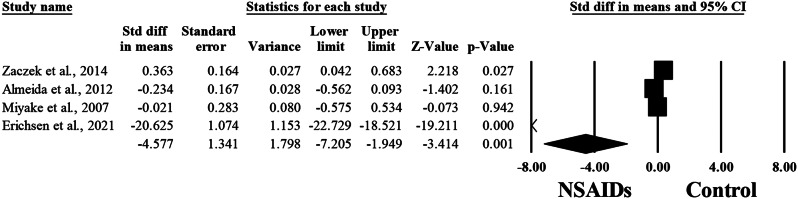
Forest plot of summary analysis of the Standardized Mean Difference (SMD) and 95% CI of the mean intraocular pressure between the NSAIDs group and control group. The size of the black squares is proportional to the statistical weight of each trial. The black diamond represents the pooled point estimate. The positioning of both diamonds and squares (along with 95% CIs) beyond the vertical line (unit value) suggests a significant outcome (IV = inverse variance)

The difference between NSAIDs and control group regarding the mean intraocular pressure was reported in four articles, including 552 patients [11, 14, 16, 27]. Pooling the data in the random-effects model (I^2^ = 99.1%, P&lt;0.001) revealed a significantly low mean intraocular pressure among patients treated with NSAIDs (SMD, -4.577; 95%CI -7.205 to -1.949; *P* = 0.001). (Fig. 8).

## Discussion

This systematic review and meta-analysis compared the effectiveness of different topical prophylactic drops on the incidence of CME following cataract surgery. The literature showed that PCME development has been linked to several variables such as light toxicity, vitreomacular traction, vascular instability, and inflammation; however, the former is considered the primary cause of PCME [1–3]. The surgical manipulation of the anterior chamber releases arachidonic acid, triggering the synthesis of inflammatory mediators. This compromises the blood-retinal barrier and results in fluid accumulation in the retinal layers [4]. The recognized mechanism of action of NSAIDs is the inhibition of both types of cyclooxygenase enzymes 1 and 2. It thereby blocks and reduces the ensuing inflammatory consequences of endoperoxide formation, particularly those of prostaglandins (28, 29, 5–6).

### Incidence of cystoid macular edema

The incidence of CME in our study is compatible with the findings of Grzybowski, who reviewed recent literature and concluded that when there are risk factors for PCME, topical NSAIDs are indicated and are useful in reducing inflammation following cataract surgery. In addition, they stated that combination therapy after surgery that contains both NSAIDs and steroids is cost-effective for healthy people [30]. This is demonstrated in PREMED report 4, where the combination group’s cost-effectiveness probability was 65%, while that of the bromfenac and dexamethasone groups was 3% and 32%, respectively [31]. Another systematic review published in 2014 found that topical NSAIDs were superior to topical steroids in reducing inflammation and incidence of PCME after simple phacoemulsification with posterior chamber intraocular lens implantation. However, the visual acuity and the incidence of adverse events were statistically unsignificant between the two group [3]. 

### Central macular thickness

We revaluated the mean difference in central macular thickness in a total of 10 studies. A statistically significant difference was found between the NSAID and other control groups. We hypothesize that intraoperative complications are the main contributors to the increased macular thickness postoperatively [3, 32]. Of the 10 studies that reported this outcome, only two had intraoperative complications. However, both studies excluded complicated cases from their analyses [17, 24]. The mean central macular thickness was found to be larger in the bromfenac group compared to the NSAID + steroid group by Wielders et al. However, at 3 months postoperatively, the mean central subfield mean macular thickness was similar [20].

### Best corrected visual acuity

Several studies assessed the difference in mean corrected visual acuity values between the NSAID and control groups, which showed that patients who received NSAID treatment had mean corrected visual acuity values that were significantly higher than those in the control group. This probably contributed to the better control of postoperative inflammation and lower incidence of PCME compared to control groups. A literature review by Kim et al. showed that prophylactic topical NSAID administration, as opposed to placebo or topical corticosteroid formulations, can decrease the incidence of CME, as determined by angiography or OCT, and might accelerate the process of visual recovery following cataract surgery [33]. However, according to level I evidence, NSAID use does not appear to lower the risk of CME-related long-term vision loss following cataract surgery [33]. In contrast, Taubenslag et al.’s results showed that corticosteroids and NSAIDs are frequently used in conjunction with cataract surgery; however, the mechanisms of action of both types of drugs overlap [34]. There is no evidence that NSAIDs improve long-term visual outcomes; however, combination therapy may hasten visual recovery [34].

### Foveal thickness

Four RCTs reported the mean change in foveal thickness. There was no significant.

difference between the NSAIDS and control groups. This could be attributed to the small sample size (*n* = 379) that studied foveal thickness pre- and postoperatively. Nevertheless, a similar result was reported by Abd El-Gawad et al., who assessed central foveal thickness using OCT and concluded that although there was no significant change in foveal thickness across both groups, the final visual outcome was similar [35]. In contrast, Duong et al. and others reported similar foveal thicknesses between the NSAIDS and steroid groups; however, the NSAID group had improved visual acuity at the 5–6-week follow-up when compared to the steroid-alone group. Although this indicates the superiority of NSAID in accelerating visual recovery, a discrepancy was created that could be explained by the inclusion of patients with DR in the study by El-Gawad et al. [33, 35, 36]. Diabetes mellitus (DM) is a special disease that requires attention. DR accounts for the increased foveal thickness in patients with DM, especially in those with proliferative DR (PDR). In this study, patients with DR were excluded; hence, no recommendations were provided [3, 36, 37]. Additionally, one RCT in this study that excluded patients with DM found that NSAID and NSAID + dexamethasone resulted in lower parafoveal thickness than dexamethasone alone. However, at 12 weeks postoperatively, all groups showed comparable parafoveal thicknesses [20].

### Intraocular pressure

In this study, the mean intraocular pressure (IOP) was evaluated in four RCTs. There was a significant difference in mean IOP between the NSAIDS and control groups., where control groups showed a statistically significant higher IOP. This is similar to the result of systematic review and meta-analysis by Kessel et al. who found a significant mean difference of 0.5 mmHg between both groups [3]. In contrast, in two recent RCTs, there was no significant difference in IOP among the NSAID, steroid, and combination groups [20, 35]. Steroids are known to cause high IOP, which gives NSAIDs the advantage of stabilizing IOP. However, the increase in IOP associated with steroid use is mild and self-limiting, as reported by the American Academy of Ophthalmology [33, 38].

The present review adds to the literature on prophylactic regimens for pseudophakic CME and shows that NSAIDs are superior in patients undergoing cataract extraction through phacoemulsification with no established ocular disease. Moreover, this study included recently published RCTs that have not been included in previous systematic reviews.

This study had certain limitations. Different drugs and doses of both NSAIDs and steroids; variable control arms, including placebo, vehicle, steroid, or NSAID; and variability in follow-up periods across the included RCTs. Another limitation is the timing variability when providing dugs. While some studies administered only preoperative prophylaxis, others gave either postoperative prophylaxis or both. All of these factors contributed to the heterogeneity observed in this meta-analysis. Additionally, some studies have shown a high risk in some domains, such as performance and detection bias.

## Conclusion

Based on this systematic review and meta-analysis, prophylactic measures including NSAID alone or in combination with steroids shows its efficacy in reducing the incidence of PCME. NSAID alone, according to the result of this study, was superior in preventing the incidence of PCME compared to the use of steroid alone or placebo. Nevertheless, multiple factors play a role in its pathophysiology, including surgical manipulation, intraoperative complications, and ocular or systemic diseases. Therefore, there is a need for standardized prophylactic protocols for each patient category (healthy patients, those with ocular disease, and those with systemic diseases). Hence, future double-blind RCTs are required.

## Electronic supplementary material

Below is the link to the electronic supplementary material.

Supplementary Material 1

## Data Availability

All data generated or analyzed in this study are included in this article. Further inquiries can be directed to the corresponding author.

## Abbreviations

PCME: Pseudophakic cystoid macular edema
NSAID: Nonsteroidal anti-inflammatory drugs
RR: Risk ratio
CI: Confidence interval
RCT: Randomized controlled trials
LogMar: Logarithm of the minimum angle of resolution
CME: Cystoid macular edema
OCT: Optical coherence tomography
PREMED: PREvention of Macular Edema after cataract surgery study
DR: Diabetic retinopathy
PDR: Proliferative diabetic retinopathy
SMD: Standardized mean difference
DM: Diabetes mellitus
