# Supplementary material for: Prophylactic regimens for the prevention of pseudophakic cystoid macular edema: systematic review and meta-analysis
Source: Int J Retina Vitreous. 2024 Oct 10;10:72. doi: 10.1186/s40942-024-00588-8 (PMC11465684; doi:10.1186/s40942-024-00588-8)

**Systematic Search of CME SR**

Date: Tuesday 18 April 2023

EMBASE 3169

MEDLINE 989

CENTRAL 503

Before removing duplicates: 4661

Number of duplicates: 1178

Total after removing duplicates: 3483

Comment: “Observation” was not used as a keyword nor mesh term.

Result folder was sent to this email: [reem.hersi3@gmail.com](mailto:reem.hersi3@gmail.com)

1. exp Macular Edema/

2. Macular Edema$.mp.

3. Post cataract surgery macular edema$.mp.

4. pseudophakic macular edema$.mp.

5. Cystoid macular edema$.mp.

6. irvine gass syndrome$.mp.

7. Anti VEGF$.mp.

8. NSAID$.mp.

9. Anti-Inflammatory Agents$.mp.

10. exp Anti-Inflammatory Agents, Non-Steroidal/

11. Non-Steroidal$.mp.

12. exp Steroids/

13. steroid$.mp.

14. exp Randomized Controlled Trial/

15. Randomized Controlled Trials$.mp.

16. exp Clinical Trial/

17. Clinical Trial$.mp.

18. exp Randomized Controlled Trial/ or exp Clinical Trial/

19. Clinical Trial$.mp.

20. 14 or 15 or 16 or 17 or 18 or 19

21. 1 or 2 or 3 or 4 or 5 or 6

22. 7 or 8 or 9 or 10 or 11 or 12 or 13

23. 20 and 21 and 22

24. remove duplicates from 23


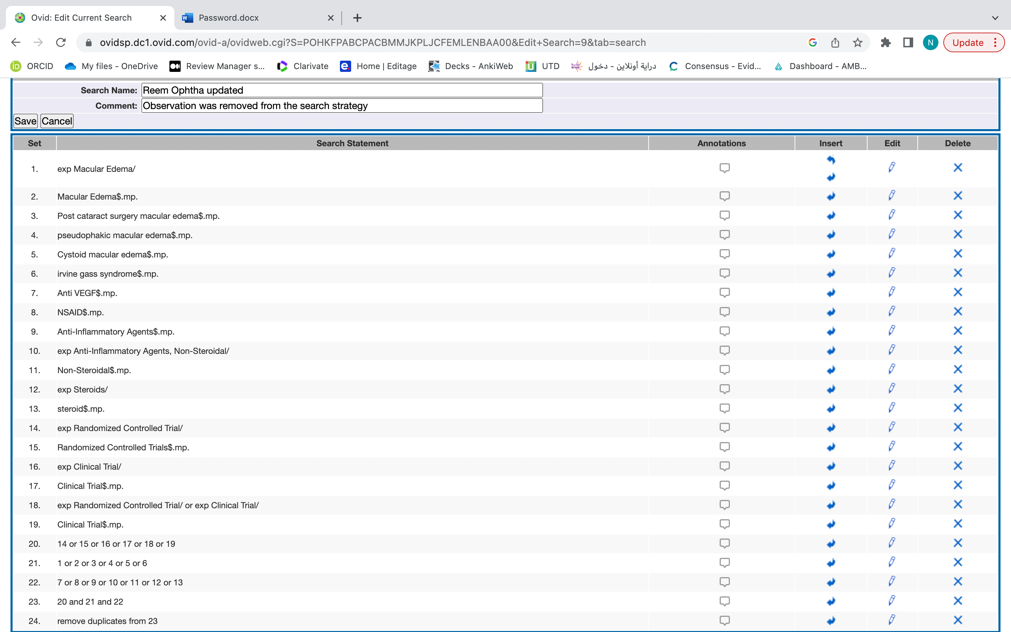

Supplement: Supplementary file 1 — Supplementary Material 1 [file 40942_2024_588_MOESM1_ESM.docx]
